# *Staphylococcus aureus* enterotoxins modulate IL-22-secreting cells in adults with atopic dermatitis

**DOI:** 10.1038/s41598-018-25125-0

**Published:** 2018-04-27

**Authors:** Raquel Leao Orfali, Luanda Mara da Silva Oliveira, Josenilson Feitosa de Lima, Gabriel Costa de Carvalho, Yasmim Alefe Leuzzi Ramos, Natalli Zanete Pereira, Naiura Vieira Pereira, Mariana Colombini Zaniboni, Mirian Nacagami Sotto, Alberto José da Silva Duarte, Maria Notomi Sato, Valeria Aoki

**Affiliations:** University of Sao Paulo Medical School, Department of Dermatology, Laboratory of Dermatology and Immunode-ficiencies (LIM-56), Sao Paulo, SP Brazil

## Abstract

Atopic dermatitis (AD) is a chronic inflammatory immune-mediated skin disease characterized by skin colonization by *Staphylococcus aureus*. Interleukin (IL)-22, in cooperation with IL-17, triggers antimicrobial peptide elaboration and enhances certain immunological responses. In AD, IL-22 is related to epidermal hyperplasia, keratinocyte apoptosis, and inhibition of antimicrobial peptide (AMP) production. We aimed to evaluate the impact of staphylococcal enterotoxins on the Tc22/Th22 induction in the peripheral blood of AD patients and on CD4^+/^CD8^+^ T cells expressing IL-22 in AD skin. Our study showed inhibition of the staphylococcal enterotoxins A and B (SEA and SEB) response by Th22 (CD4^+^IL-22^+^IL-17A^−^IFN-γ^−^) cells in AD patients. In contrast, Tc22 (CD8^+^IL-22^+^IL-17A^−^IFN-γ^−^) cells were less susceptible to the inhibitory effects of staphylococcal enterotoxins and exhibited an enhanced response to the bacterial stimuli. In AD skin, we detected increased IL-22 transcript expression and T lymphocytes expressing IL-22. Together, our results provide two major findings in response to staphylococcal enterotoxins in adults with AD: dysfunctional CD4^+^ IL-22 secreting T cells and increased Tc22 cells. Our hypothesis reinforces the relevance of CD8 T cells modulated by staphylococcal enterotoxins as a potential source of IL-22 in adults with AD, which is relevant for the maintenance of immunological imbalance.

## Introduction

Our skin barrier is a complex network with various functions, such as immunological responses, homeostasis, protection against pathogens, epidermal and adnexal systems with a perfect coordination between immune, neural and endocrine systems^1–5^. Any imbalance in this milieu, including environmental stressors, leads to several inflammatory skin conditions, mainly in atopic dermatitis^6,7^.

Atopic dermatitis (AD) is a prevalent, chronic, inflammatory, pruritic, immune-mediated skin disease^8^. Complex interactions between susceptibility genes encoding skin barrier molecules^9^, markers of the inflammatory response, environmental factors, and infectious agents, especially *Staphylococcus aureus* (*S. aureus*) and herpes virus, with aggravation by altered immunologic responses, are crucial elements for the pathophysiology of AD^10,11^.

The immune pathogenesis of AD has been described as a representative disease involving dysfunction of the Th1/Th2 biphasic immune response^12^. However, with the characterization of new subsets of human T helper (Th) cells, including Th17 and Th22^13^, novel pro-inflammatory and inflammatory components should be considered and better evaluated in AD^10,12,14^.

IL-17, which is produced by Th17 cells, is able to coordinate local tissue inflammation through the upregulation of pro-inflammatory cytokines and chemokines, including IL-6, TNF-α, IL-1β, CXCL1, CCL2, CXCL2, CCL7, and CCL20^14,15^. In cooperation with IL-17, IL-22 triggers antimicrobial peptide elaboration and enhances immunological responses^16,17^. It has been hypothesized that high IL-22 and low IL-17 expression predominate during the chronic phase of AD; therefore, the initial hypothesis of AD as a Th2-driven disease should be modified with the addition of the Th22/Tc22 subsets associated with epidermal changes^18^.

Th2 and Th22 cytokines are capable of modulating the AD epidermal barrier, including suppression of keratinocyte differentiation and apoptosis, hyperplasia, and antimicrobial peptide (AMP) production^19^. Furthermore, Th2 (IL-4, IL-13, and IL-31) and Th22 (IL-22) cytokines induce the inhibition of certain skin barrier protein-encoding genes, such as filaggrin, loricrin, and involucrin^19–21^. Th22 cells have demonstrated a capacity to infiltrate AD skin and release IL-22 and TNF-α. Likewise, significant amounts of CD3^+^CD8^+^ T cells from inflammatory skin diseases can also stimulate IL-22 secretion^22,23^. In summary, the major function of IL-22 secreting cells in AD is related to keratinocyte effects via the STAT3 (signal transducer and activator of transcription 3) pathway, enhancing cell proliferation and migration and resulting in hyperplasia, keratinocyte apoptosis, and inhibition of AMP production^19,22^.

*S. aureus* colonizes skin in approximately 30–50% of healthy adults, but it is constantly detected in only 10–20% of such individuals^24^. In AD patients, more than 90% of the skin exhibits an increase in *S. aureus* colonization as compared with the skin of healthy subjects^25^. Chronic colonization in AD skin remains a relevant issue, considering the widespread occurrence of antibiotic-resistant strains (methicillin-resistant *Staphylococcus aureus*; MRSA)^26^.

In AD patients, cutaneous *S. aureus* is capable of inducing myeloid-derived suppressor cells, leading to *in vivo* immune suppression of the T cell activation in the skin; in mice exposed to wild-type *S. aureus* on skin, there are reduced numbers of CD4^+^ and CD8^+^ T cells in the spleen^27^. Moreover, evidence of decreased peripheral blood mononuclear cell (PBMC) proliferation response to staphylococcal enterotoxin A (SEA) and other recall antigens and mitogens (TT, CMA, and PHA) suggests a compromised immune profile in adults with AD^28^.

Effector memory T cells directed against antigens derived from skin pathogens such as *S. aureus* are important for providing instant protection against viral and/or bacterial infections^29^. CD4 T cell functions include cytokine production to control infections, in addition to optimizing and maintaining CD8 T cell memory^30,31^. In this study, we aimed to evaluate the potential role of staphylococcal enterotoxins (SEA and SEB) in modulating IL-22 producing CD4^+^/CD8^+^ T cells in adults with atopic dermatitis.

## Results

### Staphylococcal enterotoxins inhibit CD4^+^ T cells secreting IL-22

In a previous study, our group demonstrated a decreased PBMC proliferation response to SEA compared with SEB stimulation, recall antigens, and mitogens in adults with AD^28^. Considering that IL-22 is beneficial to the host in many infectious and inflammatory disorders, with inherent pro-inflammatory properties^32^, we analyzed the effects of staphylococcal enterotoxins on IL-22 secretion by PBMCs in AD patients.

**Figure 1:**
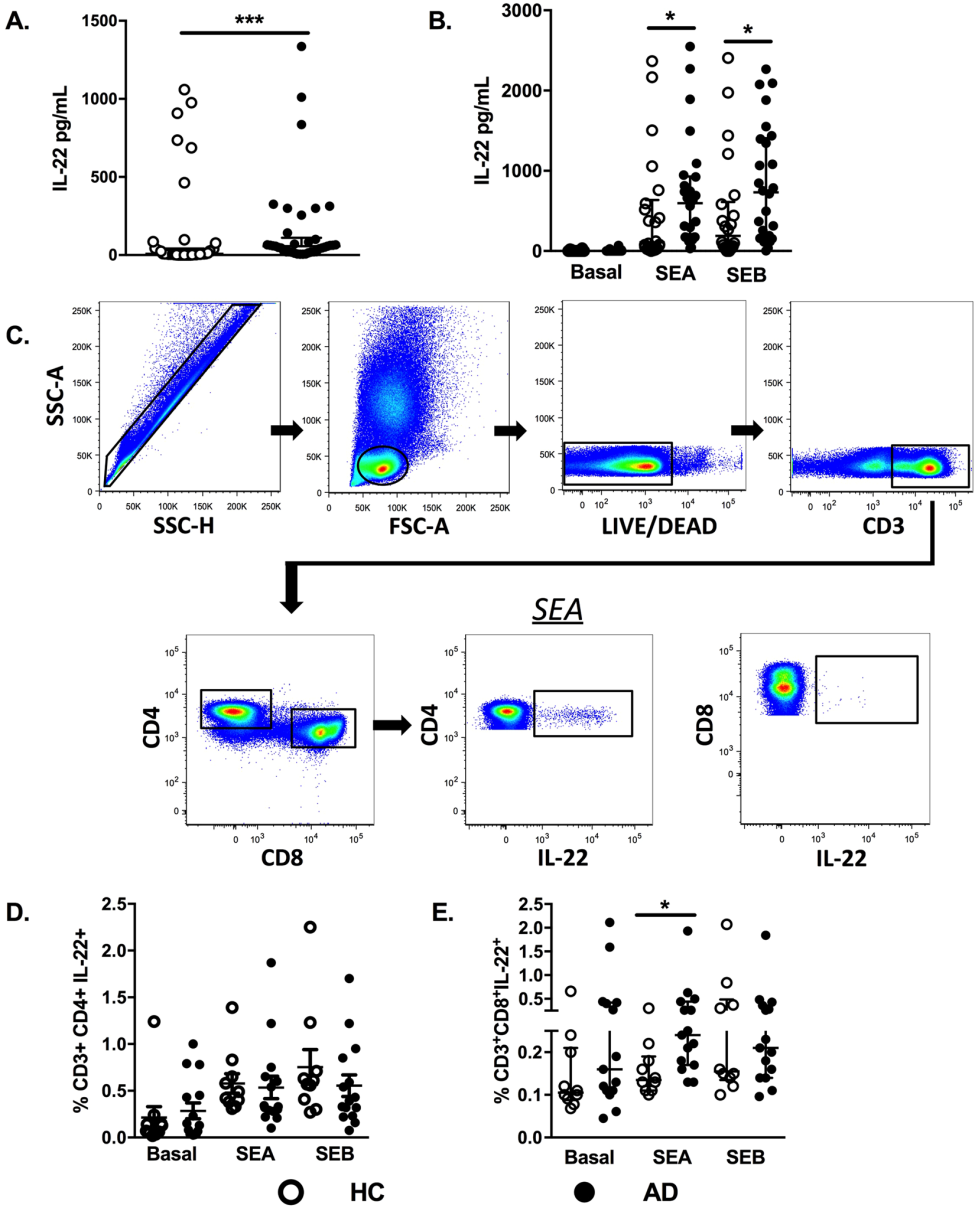
IL-22 levels in sera and PBMCs induced by SEA and SEB in AD. (**A**) Determination of IL-22 serum levels from healthy controls (HC, n = 40) and atopic dermatitis patients (AD, n = 38), assessed by ELISA. (**B**) IL-22 levels in PBMC culture supernatants from AD patients (AD, n = 26) and healthy controls (HC, n = 23), evaluated by ELISA at baseline and with SEA and SEB stimulation after 48 hours of incubation. (**C**) Representative gating strategy for the selection of populations containing CD3, CD4, and CD8 T cells. Each subsequent panel shows only the population of interest producing IL-22 after SEA and SEB stimulation. Frequencies of CD4 T cells and (**D**) CD8 T cells (**E**) secreting IL-22 after SEA and SEB stimulation from healthy controls (HC, n = 10) and adults with atopic dermatitis (AD, n = 15), evaluated by flow citometry. Data represent the medians with interquartile ranges. ^*^p ≤ 0.05 and ***p ≤ 0.001.

First, evaluating whether IL-22 levels changed in AD patients at the systemic level, we detected a significant increase of IL-22 serum levels in AD patients compared with the healthy control (HC) group (Fig. 1A). Next, IL-22 secretion induced by SEA and SEB stimulation in PBMC from AD patients and controls was assessed. Figure 1B shows a marked increase of IL-22 levels in AD patients with both SEA and SEB stimulation compared with the HC group (Fig. 1B).

The gating strategy for CD4^+^/CD8^+^ T cells producing IL-22, as analyzed by flow cytometry, is shown in Fig. 1C. The frequency of these cells was evaluated after stimulation of PBMCs with SEA and SEB. The frequency of CD4^+^IL-22^+^ T cells was decreased when compared with the controls (Fig. 1D). A different profile from that in CD4^+^ T cells was seen in CD8^+^ T cells, where IL-22 was significantly increased following SEA stimulation (Fig. 1E). Moreover, no changes in the frequencies of CD4^+^ or CD8^+^ T cells secreting IL-17 under SEA and SEB stimulation were detected in DA individuals compared with the HC group (data not shown). It is possible that IL-17-secreting cells are less refractory to the inhibitory effects of SEA/SEB. There was no relationship between disease severity and cytokine frequency (data not shown).

### Differential staphylococcal enterotoxin effects in circulating Th22 and Tc22 cells in AD patients

**Figure 2:**
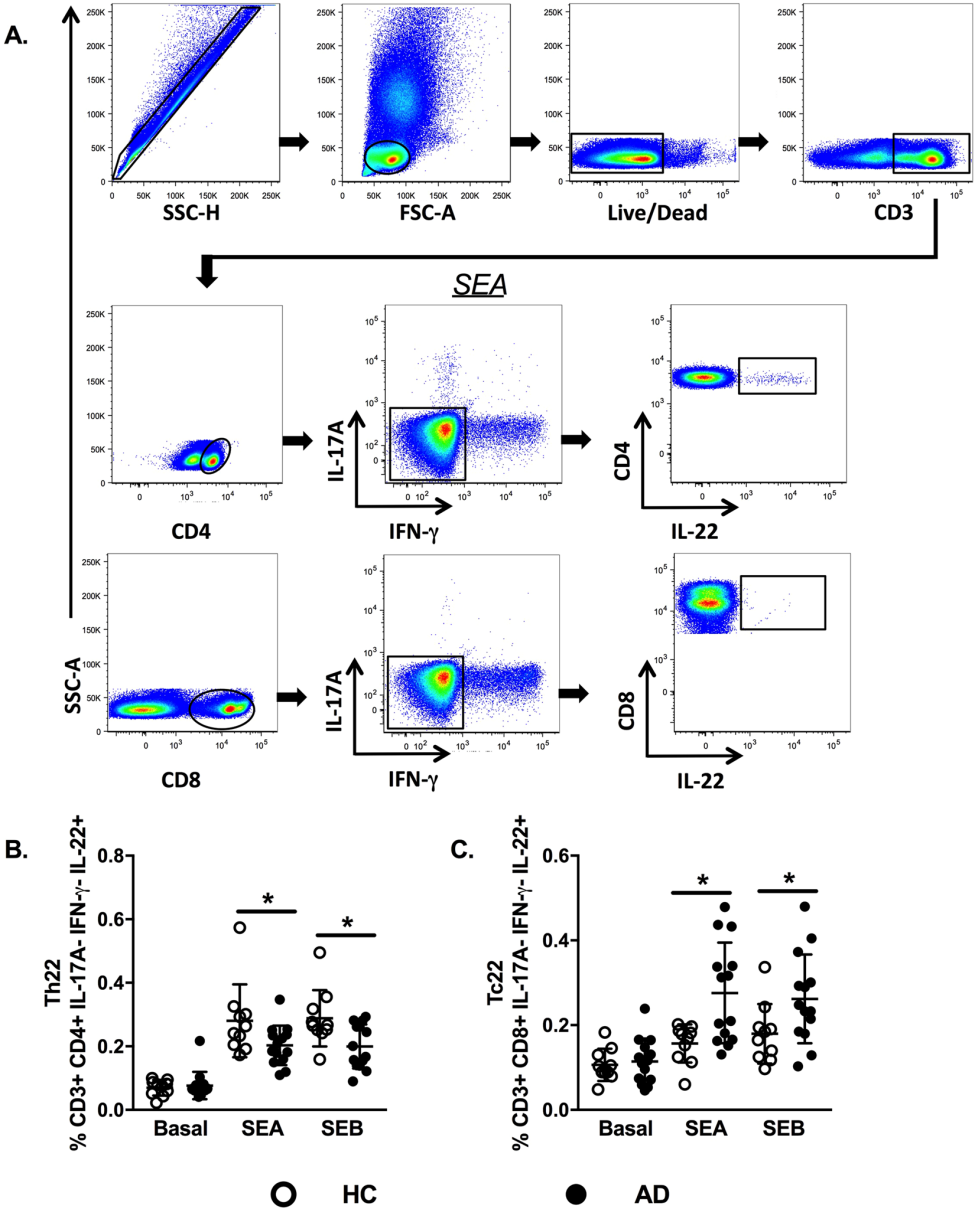
Staphylococcal antigens induce opposite effects in circulating Th22 and Tc22 cells in AD. (**A**) Gating strategy for the analysis of Th22 and Tc22 cells in PBMCs by flow cytometry, excluding IL-17A and IFN-γ secretion. (**B**) The frequencies of Th22^+^ cells represented after SEA and SEB stimulation from healthy controls (HC, n = 10) and adults with atopic dermatitis (AD, n = 15). (**C**) The frequencies of Tc22^+^ cells represented after SEA and SEB stimulation from healthy controls (HC, n = 10) and adults with atopic dermatitis (AD, n = 15). Data represent the medians with interquartile ranges. *p ≤ 0.05.

Although Th22 and Tc22 cells have been described as relevant participants in AD etiopathogenesis, more evidence is needed concerning the effects of staphylococcal enterotoxins on these T cell subsets^18,33^. Th22 cells produce large amounts of IL-22 but do not secrete IL-17A or IFN-γ^17^. To evaluate Th22 cells by flow cytometry, we excluded IL-17A^+^ and IFN-γ^+^ cells in the gating strategy, defining Th22 cells as CD3^+^CD4^+^IL-17A^−^IFN-γ^−^IL-22^+^ and Tc22 cells as CD3^+^CD8^+^IL-17A^−^IFN-γ^−^IL-22^+^ (Fig. 2A).

There was a remarkable difference between the Th22 and Tc22 cell responses in AD patients. CD4^+^IL-22^+^ T cells showed a diminished response to SEA/SEB stimuli when comparing AD patients with HC subjects (Fig. 2B). In contrast, when evaluating CD8^+^ T cells, we found augmented levels of IL-22 after stimulation in AD patients compared with those in HC subjects (Fig. 2C). These findings may indicate that staphylococcal enterotoxins have opposite effects on T cell subsets, suppressing Th22 cells and enhancing Tc22 cells in AD.

### IL-22 expression profile in skin lesions from adults with AD

Considering that there are IL-22-secreting keratinocytes and epidermal thickening with impaired barrier function in AD^20,34,35^, we then evaluated the IL-22 expression profiles in skin samples of AD patients.

**Figure 3:**
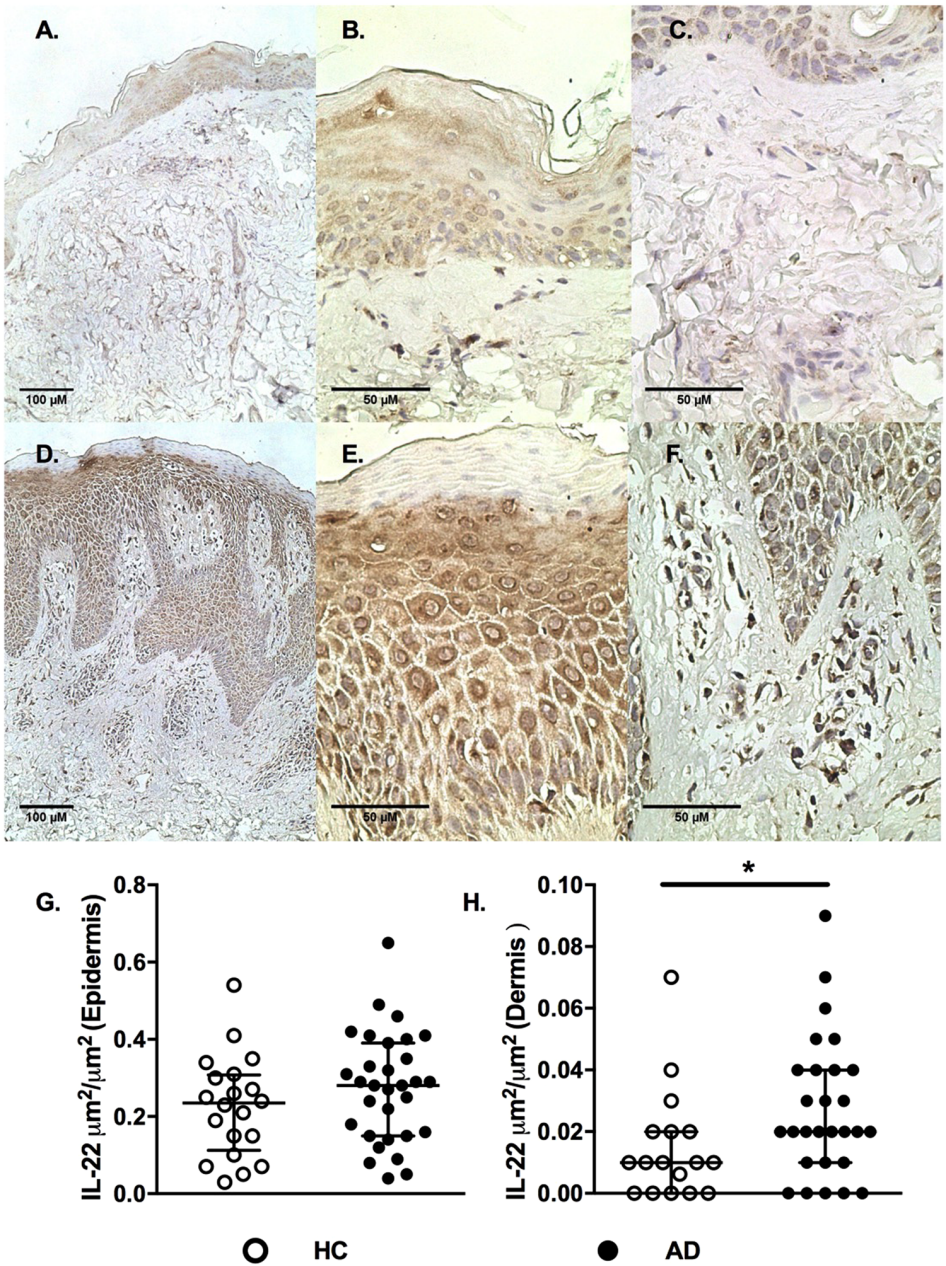
Expression of IL-22 in skin specimens from adults with AD tested by immunohistochemistry. (**A**) Photograph of a skin specimen from a healthy control: IL-22 expression. (**B**) Photograph of epidermis from a healthy control: IL-22 expression. (**C**) Photograph of dermis from a healthy control: IL-22 expression. (**D**) Photograph of a skin specimen from an AD patient: enhanced IL-22 expression. (**E**) Photograph of epidermis from an AD patient: enhanced IL-22 expression. (**F**) Photograph of dermis from an AD patient: enhanced IL-22 expression. IL-22 expression levels (μm^2^/μm^2^) in the epidermis (**G**) and dermis (**H**) of the healthy control group (HC, n = 20) compared with AD patients (AD, n = 31), evaluated by immunohistochemistry. Lines represent medians with interquartile ranges of cytokines in skin specimens. *p ≤ 0.05 and **p ≤ 0.01.

Figure 3A–C show the IL-22 expression profile in skin specimens from HCs, and Fig. 3D–F show the IL-22 expression profile in skin specimens from AD individuals. The profile of IL-22 expression of AD patients was similar to that of the controls at the epidermal level (Fig. 3G), in contrast to the enhanced dermal IL-22 expression observed in AD patients when compared with the controls (Fig. 3H).

**Figure 4:**
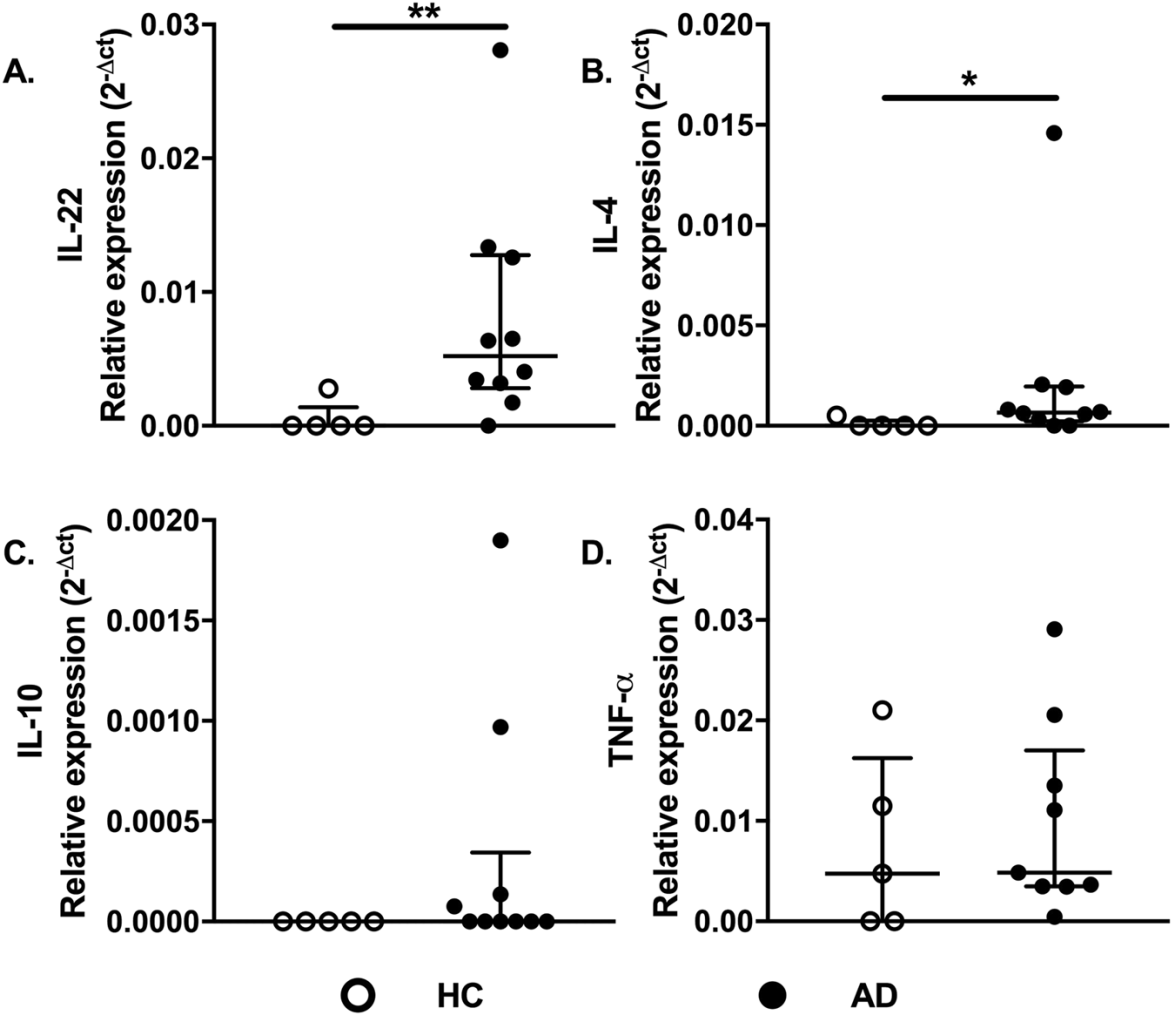
Cytokine gene expression profiles in AD skin. Expression profiles of IL-22, IL-4, IL-10, and TNF-α in skin of the healthy control group (HC, n = 5) compared with AD patients (AD, n = 10), assessed by real-time PCR. Lines represent medians with interquartile ranges of cytokines in skin specimens. *p ≤ 0.05 and **p ≤ 0.01.

**Figure 5:**
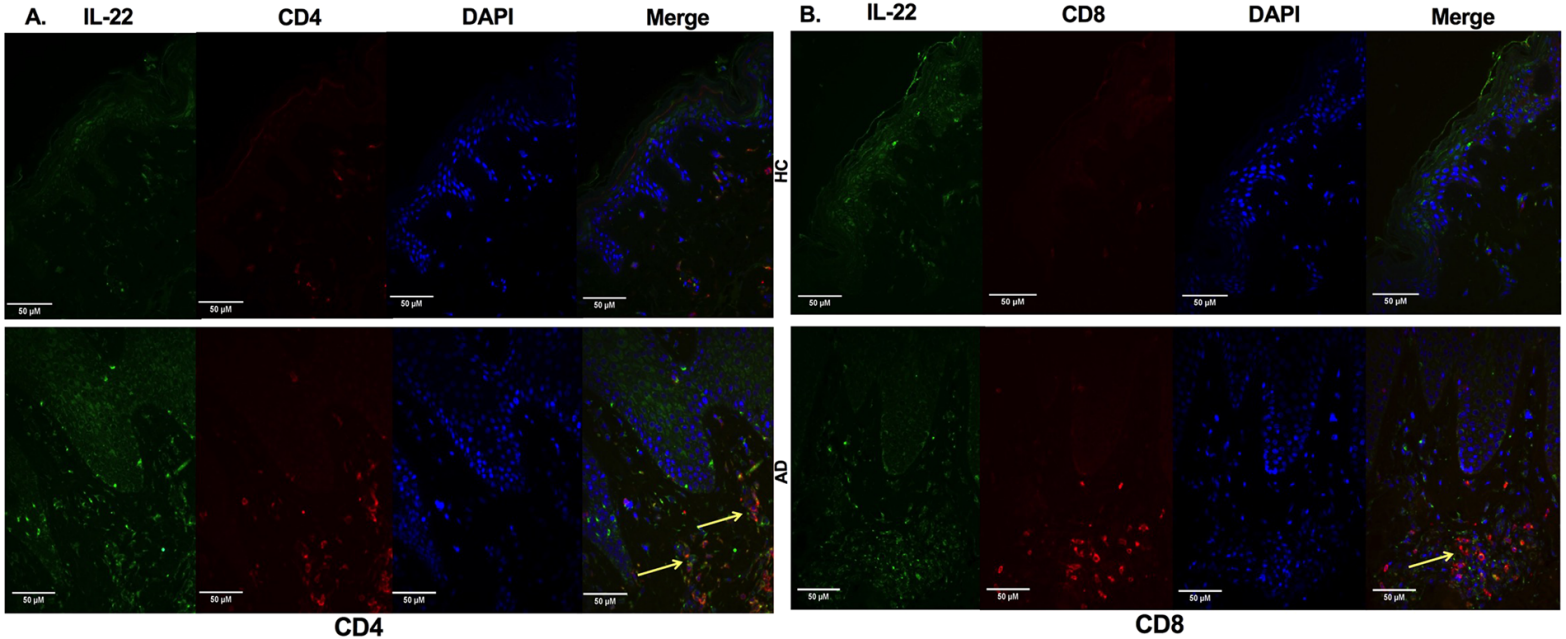
Immunofluorescence staining of CD4/IL22 and CD8/IL22 in AD skin. Double-label immunofluorescence panels showing that both CD4^+^ (**A**) and CD8^+^ dermal cells express IL-22 (**B**). Yellow arrows show the merge of CD4/CD8 T cells expressing IL-22.

We then evaluated whether IL-22 transcript levels could be altered in AD. Figure 4 shows increased expression levels of IL-22 (Fig. 4A) and IL-4 (Fig. 4B) in AD samples, with similar levels of IL-10 (Fig. 4C) and TNF-α (Fig. 4D) gene expression in the two groups. To investigate the presence of IL-22-secreting T cells, we analyzed CD4^+^/CD8^+^ T cells in skin sections of AD and HC samples (Fig. 5A,B). The enhanced IL-22 expression within the dermis in AD may have a relevant role in the immune response, considering that dermal leukocyte clusters are essential structures for eliciting acquired cutaneous immunity^36^.

## Discussion

Our study showed compromised responses of Th22 cells to SEA and SEB in the peripheral blood of AD patients, with an increase in Tc22 cells, which appear to be less susceptible to the enterotoxins’ effects. Moreover, we demonstrated the presence of CD4^+^/CD8^+^ T cells secreting IL-22 within the dermis of AD skin.

The present group of atopic patients had long-lasting AD (with a mean of 19.45 years), with increased circulating IL-22 levels and high levels secreted by PBMCs after stimulation with SEA and SEB. The main source of IL-22, a cytokine often released by inflamed tissue and detected in this study in both serum and in PBMCs, may include CD4^+^ T cells (e.g., Th17, Th22, Th1, and smaller numbers of Th2 cells)^22,34,35^, CD8^+^ T cells, natural killer cells, and dendritic cells^22,37,38^.

However, when we evaluated intracellular IL-22 expression in CD4^+^ T cells, a diminished frequency was detected, in contrast to the increased frequency of CD8^+^ T cells secreting IL-22, induced by SEA and SEB stimulation in AD patients. Interestingly, the decreased IL-22 secretion by CD4^+^ T cells is reinforced by previous data that showed inhibited statuses in both the antigen-specific proliferative response and polyclonal activators^28^ in AD, suggesting a suppressed profile of CD4^+^ T cells in AD patients, corroborated by reduced cytokine secretion after stimulation.

Other studies have shown enhanced IL-22 levels in the CD4^+^ T cells of children and adults with AD following stimulation with α-toxin from *S. aureus* and SEB^39,40^. Our cohort of adults may display a different profile compared with children due to chronic stimulation with staphylococcal enterotoxins, driving them toward a chronic activation and nonresponsive status.

Interestingly, our data indicated that *in vitro*, staphylococcal enterotoxins exert opposite effects on T cell subsets, suppressing Th22 cells and enhancing Tc22 cells after SEA and SEB stimulation in the AD group. CD4^+^IL22^+^ T cells appeared to be more susceptible than CD8^+^IL-22^+^ T cells to SEA/SEB effects, regardless disease severity. However, similar frequencies of circulating Th22 and Tc22 cells after stimulation with PMA/Ionomycin have been described in AD and psoriasis^23^. Moreover, in severe AD, selective expansion of circulating Th2/Tc2 and Th22/Tc22 cells has been described in CLA^+^ T cells induced by PMA/ionomycin^33,41^. Our results suggest that circulating Tc22 cells producing IL-22 contribute to the perpetuation of inflammation in AD patients, despite the presence of IL-17 and IFN-γ, reinforcing the relevance of CD8^+^ T cells modulated by staphylococcal enterotoxins in AD pathogenesis.

In AD dermal lesions, we detected enhanced expression of IL-22; additionally, we identified both CD4 and CD8 T cells expressing IL-22 in AD skin, in accordance with previous findings^42,43^. These authors found dermal cellular infiltrates of mainly CD4^+^ and CD8^+^ T cells, with a CD4:CD8 ratio similar to the profile detected in the peripheral blood in AD subjects^42,43^. Furthermore, high percentages of CD8^+^ T cells isolated from AD skin explant cultures are related to IL-22, IFN-γ, IL-13, and IL-17 production^44^, reinforcing the important role of these cells in barrier dysfunction and as a significant source of inflammatory cytokines in AD^23^.

We also described increased IL-22 and IL-4 transcript expression, emphasizing the Th2/T22 deviation in AD patients. Epicutaneous sensitization to house dust mites induces upregulation of IL-22 in an AD-like skin mouse model, leading to pruritus, intensified dermal and systemic Th2 immunity, downregulation of epidermal differentiation complex genes, and enhanced epidermal colonization of *S. aureus*^45^. In AD, decreases in the skin barrier function and Th2 cytokine release favor *S. aureus* penetration of the skin, with diminished *in vitro* expression of filaggrin and human β-defensin (HBD) 3^46,47^.

In conclusion, staphylococcal enterotoxins may play a role as a modulating factor on T lymphocyte–IL-22 secreting cells in AD patients, as evidenced by the presence of CD4/CD8 T cells expressing IL-22 in skin lesions, dysfunctional circulating Th22, and Tc22 cell upregulation.

## Methods

### Subjects

Thirty-eight patients with AD (aged between 19–48 years; mean age: 27.68 ± 8.45; 26 males and 12 females) and 40 healthy non-AD volunteers (aged between 19–53 years; mean age: 31.10 ± 9.05; 18 males and 22 females) were enrolled in this study. AD was diagnosed according to the Hanifin & Rajka criteria^48^. Disease severity was evaluated by the EASI (Eczema Area and Severity Index)^49^, and AD patients were classified as mild (n = 9), moderate (n = 15), or severe (n = 14). IgE serum levels ranged from 3,120 to 119,000 IU/mL (average of 29,681). None of the patients were using oral steroids or immunosuppressants. All patients were interviewed about any associations with respiratory symptoms and the age of AD onset. This study was approved by the Ethics Committee of the University of Sao Paulo School of Medicine, and informed consent was obtained from all subjects. All methods were performed in accordance with the relevant guidelines and regulations of this institution. Demographic data are shown in Tables 1 and S1 (supplementary file).

**Table 1:** Demographic data of adults with AD.

Identification	Gender	Age	EASI	IgE (IU/mL)	Eosinophils %
PC1	M	30	48.4	18300	6.6
PC2	F	22	25	40100	8.2
PC3	F	29	40.6	59500	19.4
PC4	M	21	47.7	39100	11.3
PC5	F	43	33.5	2680	5.7
PC6	M	28	40.2	16700	6.4
PC7	M	24	47.9	3120	10.1
PC8	F	22	10.3	26200	7.8
PC9	M	37	47.4	59900	5.1
PC10	M	43	18.4	28300	12.7
PC11	F	22	31.6	40100	8.2
PC12	F	24	49	21200	5.2
PC13	M	22	17.8	3400	8.7
PC14	M	24	50	46300	22
PC15	M	39	35.4	5080	6.7
PC16	M	20	24.7	26900	36.1
PC17	M	19	25.9	67500	6.4
PC18	M	34	35.8	5530	13.2
PC19	M	24	35	30000	15.2
PC20	M	20	14.4	4910	7.6
PC21	M	21	48.2	54200	9.9
PC22	M	28	47.1	23300	9.1
PC23	M	31	41.9	23800	25
PC24	M	29	11.2	4070	14.2
PC25	M	21	38.5	2870	6.8
PC26	F	42	8.6	5180	9.9
PC27	M	27	51	37600	5.1
PC28	M	19	22	4140	5.9
PC29	F	28	70	45900	14
PC30	M	24	45.8	13100	12
PC31	F	48	8.7	343	1.1
PC32	M	48	41.4	34800	14.9
PC33	F	20	34.8	119000	5.5
PC34	F	25	31.2	13400	9.2
PC35	M	28	35.2	5260	6.6
PC36	M	19	35.2	58400	24.2
PC37	F	31	12	3400	6.9
PC38	M	19	21.4	6050	10.7
HC (n = 40)	18 M/22 F	31.1 (18–53)	NA	<100	<5

PC = patients; M = male; F = female; EASI = *eczema area and severity index*; AD = atopic dermatitis; HC = healthy controls; NA = not applicable.

### ELISA

PBMCs were isolated from heparinized venous blood via Ficoll-Hypaque gradient centrifugation (GE Healthcare Bio-Sciences AB, Uppsala, Sweden) and resuspended in RPMI 1640 medium supplemented with gentamicin (40 µg/mL) and 10% pooled AB normal human serum (Sigma-Aldrich, St. Louis, MO, USA). PBMCs (2 × 10^6^/well) were cultured in 96-well microplates (Costar, Cambridge, MA, USA) at 2 × 10^5^ cells/well with SEA (0.04 µg/mL; Sigma) and SEB (1 µg/mL; Sigma) for 48 hours at 37 °C in 5% CO_2_. Serum samples were also obtained. Cell-free supernatants and serum samples were stored at −70 °C until use in cytokine assays. IL-22 measurements were performed by ELISA in both culture supernatants and sera, following the manufacturer’s recommendations (DY782, R&D Systems, Minneapolis, MN, USA). The detection limit of IL-22 was 31.2 pg/mL.

### Flow cytometry

PBMCs (2 × 10^6^/well) were cultivated in 96-well microplates (Costar) at 37 °C and 5% CO_2_ in the presence of SEA (0.04 µg/mL; Sigma) and SEB (1 µg/mL; Sigma), for 6 hours. Brefeldin A (10 µg/mL; Sigma) was added 2 hours after the culture was started. Next, the cells were stained with the viability marker LIVE/DEAD PE-Texas Red (Invitrogen, Carlsbad, CA, USA) for 30 minutes at room temperature. Cells were then incubated with Cytofix/Cytoperm (BD Biosciences, San Jose, CA, USA) and stained with the following antibodies: CD3-Qdot 605 (Invitrogen), CD4-Horizon V500 (BD), CD8-PerCP-Cy5.5 (BD), IL-17A-Alexa 488 (eBioscience, San Diego, CA, USA), IL-22-PE (eBioscience), and IFN-γ-Horizon V450 (BD Biosciences). Cells were then fixed with 1% paraformaldehyde, and 400,000 events were acquired on an LSR Fortessa flow cytometer (BD Biosciences). Fluorescence minus one (FMO) controls were performed for all antibody panels to check for proper compensation and to define positive signals. Data analysis was performed using FlowJo v10 software (Tree Star, Ashland, OR, USA).

### Immunohistochemistry

Immunohistochemistry was performed on 4-μm paraffin-embedded samples, as described elsewhere^50^. The primary antibody IL-22 (ab134035, Abcam, Cambridge, UK) was utilized at 1:200 dilution, and the detection system was a Novolink Max Polymer Detection System (RE7280-K, Leica Biosystems, Newcastle Upon Tine, UK), and the chromogen used was DAB (3,3′ diamibenzidine, D5637, Sigma). Total tissue distribution of IL-22 was calculated by dividing the stained area by the total area measured in the epidermis or dermis. Immunohistochemically stained specimens were scanned using an Aperio Scan-scope Cs (Aperio Technologies, Vista, CA, USA). Photographs were analyzed utilizing Image-Pro Plus version 4.5.0.29 (Media Cybernetics Inc., Bethesda, Maryland, USA)^51^.

### Immunofluorescence assay

Immunofluorescence was performed on 4-μm paraffin-embedded samples, as described elsewhere^50^. After dewaxing, samples were fixed with paraformaldehyde 4% and blocked with phosphate-buffered saline with bovine serum albumin (PBS-BSA) 2% for 30 minutes. The primary antibodies IL-22 (ab134035, Abcam), CD4 (104R-16, Cell Marque, Rocklin, CA, USA), or CD8 (ab101500, Abcam) were incubated overnight at 4 °C. On the following day, the secondary antibodies, donkey anti-mouse Alexa 488 fluorescein (A21202, ThermoFisher Scientific, Waltham, MA, USA) (for IL-22 staining) and goat anti-rabbit Alexa 546 fluorescein (A11035, ThermoFisher Scientific) (for CD4 or CD8 staining), and 4′,6-diamidino-2-phenylindole dihydrochloride (DAPI, D1306, ThermoFisher Scientific) for nuclear identification were diluted and incubated for 90 minutes at room temperature. Images were acquired utilizing the appropriate filters of an Axiovert 200 inverted immunofluorescence microscope (Zeiss, Oberkochen, Germany).

### Real-time PCR

Six-millimeter punch samples were taken from the skin lesions of AD patients with local anesthesia. Normal skin samples as controls were obtained after plastic surgery. All of the specimens were stored in RNAlater solution (Sigma), at −20 °C. Frozen samples were cut, and the tissue was homogenized with a Tissue Ruptor (Qiagen, Valencia, CA, USA) after debris removal by centrifugation, and supernatants were used for RNA extraction. Total RNA was extracted from each skin biopsy using an RNeasy Plus Mini Kit (Qiagen), including an extra step for separation of genomic DNA (gDNA eliminator column). The samples were then treated with a DNase set (Qiagen). Reverse transcription was performed with a Reverse Transcriptase Kit (Bio-Rad, Hercules, California, USA). For PCR amplification, TNF-α gene: forward primer (5′-CCCAGGCAGTCAGATCATCTTC-3′) and reverse primer (5′-GCTTGAGGGTTTGCTACAACAT-3′); IL-4 gene: forward primer (5′-CCAACGTACTCTGGTTGGCT-3′) and reverse primer (5′-GCACCGAGTTGACCGTAACA-3′); IL-10 gene: forward primer (5′- CACATGCGCCTTGATGTCTG-3′) and reverse primer (5′-CAGGGCACCCAGTCTGAGA-3′); IL-22 gene: forward primer (5′-GAGAAACTGTTCCACGGAG-3′) and reverse primer (5′-TGCTTAGCCTGTTGCTGAG-3′); and GAPDH gene: forward primer (5′-GAAGGTGAAGGTCGGAGT-3′) and reverse primer (5′-GAAGATGGTGATGGGATTTC-3′) were utilized.

Real-time PCR was performed in an Applied Biosystems 7500 system using specific primers and SYBR Green (Applied Biosystems, Carlsbad, CA, USA), as described by Pereira *et al*.,^52^. Primers for qPCR (Life Technologies) were only accepted if their efficiency reached 100 ± 10%. Corrections were made for primer efficiency. The specificity of the reaction was examined by dissociation curve. Glyceraldehyde-3-phosphate dehydrogenase (GAPDH) mRNA levels of the samples in the same plate were analyzed to normalize the mRNA contents among the tested samples. The cycling protocol consisted of 10 minutes at 95 °C, followed by 40 cycles of 15 seconds at 95 °C and 60 seconds at 60 °C. The amplification results were analyzed using Sequence Detection System (SDS) software (Applied Biosystems). Normalized expression was calculated as previously described by Livak^53^.

### Statistical analysis

The Mann–Whitney test or Kruskal–Wallis with Dunn’s post-test were utilized to compare 2 or 3 sets of data, respectively. Correlations were established using the Spearman non-parametric correlation test. Differences between groups were considered statistically significant when p ≤ 0.05.

## Electronic supplementary material

Dataset 1
