# Supplementary material for: Staphylococcus aureus enterotoxins modulate IL-22-secreting cells in adults with atopic dermatitis
Source: Sci Rep. 2018 Apr 27;8:6665. doi: 10.1038/s41598-018-25125-0 (PMC5923268; doi:10.1038/s41598-018-25125-0)
Supplement: Supplementary file 1 — Dataset 1 [file 41598_2018_25125_MOESM1_ESM.docx]

**Title: *Staphylococcus aureus* enterotoxins modulate IL-22-secreting cells in adults with atopic dermatitis**

**Authors:** Raquel Leao Orfali*, Luanda Mara da Silva Oliveira, Josenilson Feitosa de Lima, Gabriel Costa de Carvalho, Yasmim Alefe Leuzzi Ramos, Natalli Zanete Pereira, Naiura Vieira Pereira, Mariana Colombini Zaniboni, Mirian Nacagami Sotto, Alberto José da Silva Duarte, Maria Notomi Sato^+^, Valeria Aoki^+^.

University of Sao Paulo Medical School, Department of Dermatology, Laboratory of Dermatology and Immunodeficiencies (LIM-56) - Sao Paulo-SP – Brazil

^+^These authors shared the mentorship, critical revision and supervision of this study.

*Corresponding author: Raquel Leao Orfali, MD, PhD.

Department of Dermatology, University of Sao Paulo School of Medicine

Address: Av. Dr. Enéas de Carvalho Aguiar, 255, 3o. andar ICHC, Sala 3016, Cerqueira César, São Paulo-SP-Brazil. CEP- 05403-002. E-mail: raquelleao@hotmail.com

| **Supplementary Table S1 - Demographic data of healthy control group** | | | | |
| --- | --- | --- | --- | --- |
| **Identification** | **Gender** | **Age** | **IgE (IU/mL)** | **Eosinophils %** |
| HC1 | F | 21 | <100 | <5.0 |
| HC2 | F | 35 | <100 | <5.0 |
| HC3 | M | 29 | <100 | <5.0 |
| HC4 | F | 20 | <100 | <5.0 |
| HC5 | F | 21 | <100 | <5.0 |
| HC6 | M | 31 | <100 | <5.0 |
| HC7 | M | 26 | <100 | <5.0 |
| HC8 | F | 25 | <100 | <5.0 |
| HC9 | M | 24 | <100 | <5.0 |
| HC10 | F | 43 | <100 | <5.0 |
| HC11 | F | 25 | <100 | <5.0 |
| HC12 | M | 30 | <100 | <5.0 |
| HC15 | M | 29 | <100 | <5.0 |
| HC14 | M | 25 | <100 | <5.0 |
| HC13 | M | 28 | <100 | <5.0 |
| HC16 | F | 27 | <100 | <5.0 |
| HC17 | M | 53 | <100 | <5.0 |
| HC18 | M | 31 | <100 | <5.0 |
| HC19 | M | 43 | <100 | <5.0 |
| HC20 | M | 46 | <100 | <5.0 |
| HC21 | M | 33 | <100 | <5.0 |
| HC22 | F | 30 | <100 | <5.0 |
| HC23 | F | 26 | <100 | <5.0 |
| HC25 | F | 25 | <100 | <5.0 |
| HC24 | M | 31 | <100 | <5.0 |
| HC26 | F | 51 | <100 | <5.0 |
| HC27 | F | 52 | <100 | <5.0 |
| HC28 | F | 19 | <100 | <5.0 |
| HC29 | F | 22 | <100 | <5.0 |
| HC32 | F | 28 | <100 | <5.0 |
| HC30 | F | 41 | <100 | <5.0 |
| HC31 | F | 40 | <100 | <5.0 |
| HC33 | M | 29 | <100 | <5.0 |
| HC34 | F | 41 | <100 | <5.0 |
| HC35 | M | 24 | <100 | <5.0 |
| HC38 | F | 26 | <100 | <5.0 |
| HC36 | F | 38 | <100 | <5.0 |
| HC37 | M | 24 | <100 | <5.0 |
| HC39 | M | 26 | <100 | <5.0 |
| HC40 | F | 26 | <100 | <5.0 |
| HC = healthy control; M = male; F = female | | | | |
